# Developmental dysplasia of the hip alters fracture patterns in femoral neck fractures

**DOI:** 10.1038/s41598-026-51920-1

**Published:** 2026-05-05

**Authors:** Yoshio Nishida, Yuki Ogawa, Tomohiro Shimizu, Shun Shimodan, Hotaka Ishizu, Norimasa Iwasaki

**Affiliations:** 1 Department of Orthopedic Surgery, Faculty of Medicine and Graduate School of Medicine, Hokkaido University, Kita-15, Nishi-7, Kita-ku, Sapporo, 060-8638 Japan; 2 Orthopaedic Trauma Center, Sapporo Higashi Tokushukai Hospital, Sapporo, Japan; 3 Department of Orthopaedic Surgery, Kushiro City General Hospital, Kushiro, Hokkaido Japan; 4 Department of Orthopaedic Surgery, Iwamizawa Hokushokai Hospital, Iwamizawa, Hokkaido Japan

**Keywords:** Femoral neck fracture, Pauwels classification, Developmental dysplasia of the hip, Acetabular version, Pelvic morphology, Fracture pattern, Anatomy, Diseases, Health care, Medical research, Risk factors

## Abstract

Femoral neck fractures are commonly classified using Pauwels’ system, which reflects fracture-line inclination and mechanical stability. However, anatomical factors influencing fracture configuration remain unclear. This multicenter prospective cohort study investigated whether pelvic morphology, particularly developmental dysplasia of the hip (DDH), is associated with variations in Pauwels classification. Between April and December 2023, 254 surgically treated femoral neck fractures from nine hospitals were analyzed. Radiological parameters, including center–edge angle, sharp angle, acetabular roof obliquity, acetabular version (AV), and acetabular and femoral head diameters, were measured. DDH was diagnosed using established radiographic criteria. Logistic regression analyses were performed to identify independent predictors of Pauwels type I and III fractures. DDH was present in 23.6% of patients and was significantly associated with a higher proportion of Pauwels type I fractures, whereas absence of DDH and reduced AV were associated with Pauwels type III fractures. Multivariate analysis confirmed DDH as an independent predictor of Pauwels type I fractures and decreased AV as an independent predictor of Pauwels type III fractures. These findings suggest that pelvic morphology, including mild dysplasia and acetabular retroversion, contributes to fracture configuration in femoral neck fractures and may provide insight into the biomechanical mechanisms underlying fracture patterns.

## Introduction

Hip fractures represent one of the most common orthopedic injuries, particularly among the elderly. In 2019, over 10 million cases of hip fractures were reported worldwide, posing a significant burden on healthcare systems globally^1^. As life expectancy continues to increase, the global incidence of hip fractures is projected to nearly double between 2018 and 2050^2^. Femoral neck fractures, a distinct subtype of hip fractures, are classified using several systems, among which Pauwels’ classification is widely accepted. This system categorizes fractures based on the inclination angle of the fracture line relative to the horizontal plane and serves as a predictor of shear forces at the fracture site, as well as the risk of loss of reduction and nonunion^3–5^. While Pauwels type III fractures—characterized by an angle of ≥ 50°—are typically associated with high-energy trauma in younger patients^6,7^, surgeons have occasionally encountered Pauwels type III fractures resulting from low-energy mechanisms in the elderly. Several factors have been shown to influence Pauwels’ classification, including age-related changes in bone quality, such as osteoporosis and alterations in bone geometry; however, limited data exist regarding specific anatomical or biomechanical risk factors that determine Pauwels’ classification. At least two mechanisms are involved in femoral neck fractures: direct impact from a sideways fall and femoroacetabular impingement^8^. Given this, hip morphology can aid in determining fracture occurrence, fracture type, and classification.

Developmental dysplasia of the hip (DDH), a common morphological hip abnormality, is prevalent in Japan^9^. The center–edge angle (CEA) is widely used as a diagnostic measure for DDH. Yamauchi et al. reported that patients with greater CEA values are more likely to sustain intertrochanteric fractures rather than femoral neck fractures^10^, suggesting that acetabular coverage alters the biomechanical forces transmitted through the hip joint and influences fracture patterns.

Based on these observations, we hypothesized that pelvic morphology, specifically the degree of acetabular coverage, influences the fracture configuration and angles in femoral neck fractures. To our knowledge, no previous multicenter study has investigated this association specifically in femoral neck fractures, despite the high clinical relevance of fracture morphology. The primary aim of this study was therefore not to evaluate treatment strategies or clinical outcomes, but to clarify the underlying morphological mechanism that determines fracture patterns, with a focus on Pauwels classification. To address this research question, we conducted a multicenter prospective cohort study using standardized radiographic and computed tomography measurements to objectively evaluate the association between pelvic morphology and femoral neck fracture patterns.

## Methods

### Study design and patients

Our study was performed in accordance with the relevant guidelines of Hokkaido University Hospital and was approved by its Research Ethics Review Committee. Our research protocol for human samples used in this study was approved by the Research Ethics Review Committee of Hokkaido University Hospital (approval ID: 0023–0037), and informed consent was obtained from all participants. In this multicenter prospective cohort study, we enrolled patients with femoral neck fractures who underwent surgical treatment at nine hospitals between April and December 2023. This study was approved by the institutional review board of our hospital and the respective ethics committees of all participating centers. Written informed consent was obtained from all patients prior to enrollment. Demographic data collected included age, sex, body mass index (BMI), and the side of injury.

### Radiological evaluation

Preoperative radiographic evaluation was performed using anteroposterior (AP) radiographs. The center–edge angle (CEA) was defined as the angle between a vertical line passing through the center of the femoral head and a line drawn from the center of the femoral head to the lateral edge of the acetabulum. The sharp angle was measured as the angle between the line connecting the lateral aspect of the weight-bearing zone and the inferior point of the teardrop, and a line parallel to the transverse axis of the pelvis.

**Fig. 1:**
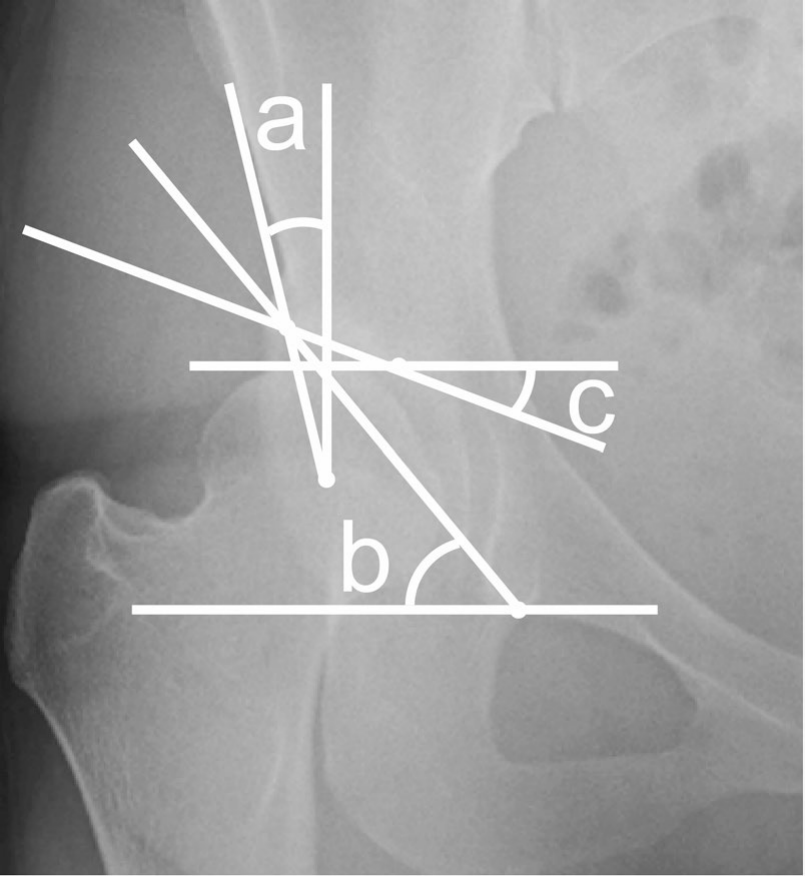
Definition of the radiological parameters. Center edge angle (**a**) is defined as the angle between the perpendicular line through the center of the femoral head and the line from the center of the femoral head to the edge of the acetabulum. Sharp angle is defined as the angle between the line joining the lateral aspect of the weight-bearing zone and the inferior point of the teardrop and the line parallel to the transverse axis of the pelvis (**b**). Acetabular roof obliquity is defined as the angle between the line joining the medial and lateral aspects of the weight-bearing zone and the line parallel to the transverse axis of the pelvis (**c**).

The acetabular roof obliquity (ARO) was defined as the angle between the line connecting the medial and lateral aspects of the weight-bearing zone and a line parallel to the transverse pelvic axis (Fig. 1). DDH was diagnosed based on the following criteria: (1) CEA ≤ 20°, (2) sharp angle ≥ 45°, or (3) ARO ≥ 15°.

**Fig. 2:**
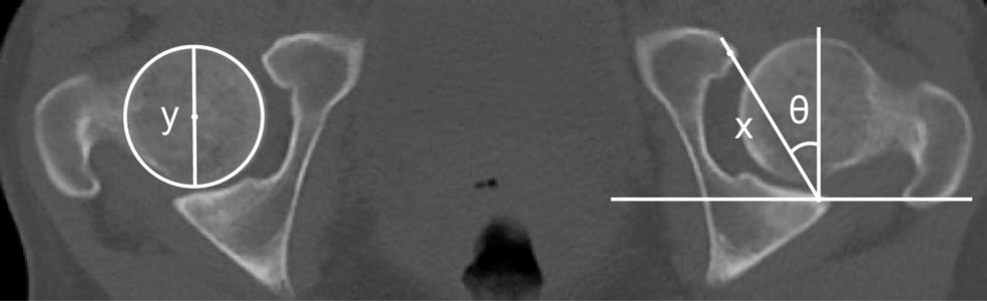
Acetabular version (AV) and diameter of the acetabulum (x) and femoral head (y) evaluated using CT axial slice through the center of the femoral head. AV was defined as the angle between the acetabular line and the perpendicular to the horizontal line (θ).

Additionally, Pauwels’ classification, Garden classification (displaced or non-displaced), and Kellgren-Lawrence (KL) osteoarthritis grade were evaluated. To minimize the influence of lower limb positioning during radiographic acquisition, the Pauwels’ angle was measured as the angle between the fracture line and a line perpendicular to the proximal femoral shaft axis^5^. Computed tomography (CT) images at the level of the femoral head center were assessed to measure acetabular version (AV), defined as the angle between the acetabular line and the perpendicular to a horizontal reference line. The diameters of the acetabulum and femoral head were measured (Fig. 2). All measurements were performed manually by two trained orthopedic surgeons (N.S. and H.I). Interobserver reliability was assessed using the intraclass correlation coefficient (ICC).

### Statistical analysis

Categorical variables were analyzed using Fisher’s exact test. Continuous variables were assessed using one-way analysis of variance (ANOVA) followed by Tukey’s multiple comparison test for comparisons among three groups and independent *t*-tests for comparisons between two groups. Univariate and multivariate logistic regression analyses were performed to calculate odds ratios (OR) and 95% confidence intervals (CI) for Pauwels type I and III fractures. Multivariate models were adjusted for significant covariates identified in the baseline analysis. The selection of adjustment variables was determined based on sample size considerations and finalized using the Akaike Information Criterion (AIC) to ensure the most robust model fit. All statistical analyses were performed using GraphPad Software (La Jolla, CA, USA), with a significance level set at *P* < 0.05. A post hoc power analysis demonstrated that, for an alpha level of 0.05, a power of 0.99 was achieved with a sample size of 254 hips for the contingency table analysis of Pauwels’ classification.

## Results

### Patients and their characteristics

A total of 254 patients with femoral neck fractures were enrolled, of whom 60 (23.6%) were diagnosed with DDH. Notably, all DDH cases in this cohort were classified as Crowe type I, indicating reduced acetabular coverage without significant proximal migration of the femoral head. The proportion of female patients was significantly higher in the DDH than non-DDH groups (86.7% vs. 70.1%, *P* = 0.01). No significant differences were observed between the groups in terms of mean age, BMI, or the side of injury (Table 1).

**Table 1:** Patient demographic characteristics.

	DDH (n = 60)	non-DDH (n = 194)	P Value
Mean age, years old	79.9 (10.9)	79.1 (11.3)	0.63
Sex, female: male	52: 8	136: 58	0.01*
Body mass index, kg/m2	21.6 (6.7)	20.8 (3.6)	0.27
Injured side, right: left	32: 28	104: 90	> 0.99

Data are presented as mean (standard deviation). *p < 0.05 indicates a statistically significant difference between the two groups.

### Comparison of radiological evaluation between patients with and without DDH

Radiological findings for patients with and without DDH are presented in Table 2. A significant difference in Pauwels’ classification was observed between the two groups, with the DDH group demonstrating a higher proportion of Pauwels type I fractures and the non-DDH group showing a higher proportion of Pauwels type III fractures (P = 0.01). In addition, the acetabular diameter (49.8 vs. 50.9 mm, *P* = 0.04) and femoral head diameter (43.3 vs. 44.4 mm, *P* = 0.03) were significantly smaller in the DDH than non-DDH groups. No significant differences were found between the groups in Garden classification, KL OA grade, or AV.

**Table 2:** Comparison of radiological evaluation between patients with and without DDH.

	DDH (n = 60)	non-DDH (n = 194)	P Value
Pauwels’ classification, cases			0.01*
I	10 (16.7%)	12 (6.2%)	
II	41 (68.3%)	126 (64.9%)	
III	9 (15.0%)	56 (28.9%)	
Garden classification, cases			0.58
non-displaced	14 (23.3%)	38 (19.6%)	
displaced	46 (76.7%)	156 (80.4%)	
Kellgren-Lawrence OA grade, cases			0.40
0	18 (30.0%)	77 (39.7%)	
1	36 (60.0%)	98 (50.5%)	
2	6 (10.0%)	19 (9.8%)	
Acetabular version, °	19.6 (7.8)	20.1 (6.3)	0.59
Diameter of acetabulum, mm	49.8 (3.5)	50.9 (3.7)	0.04*
Diameter of femoral head, mm	43.3 (3.3)	44.4 (3.5)	0.03*

Data are presented as mean (standard deviation) or case number (percentage). For categorical variables (Pauwels classification, Garden classification, and KL grade), p-values represent the results of the chi-square test or Fisher’s exact test for the overall distribution between the two groups. Continuous variables were compared using the independent t-test. *p < 0.05 indicates a statistically significant difference between the two groups.

### Comparison of radiological parameters among Pauwels’ classification

Table 3 summarizes the comparison of radiological parameters across Pauwels’ classifications. A significant decrease in AV was observed in Pauwels type III fractures compared with Pauwels type I (*P* < 0.05) and II (*P* = 0.03) fractures. However, no significant differences were detected among the groups in terms of CEA, sharp angle, ARO, acetabular diameter, or femoral head diameter. Interobserver reliability for the radiographic measurements was good to excellent. The intraclass correlation coefficients were 0.84 for the CEA, 0.88 for the Sharp angle, 0.84 for the ARO, 0.87 for acetabular version, 0.85 for the diameter of the acetabulum, and 0.87 for the diameter of the femoral head.

**Table 3:** Comparison of radiological evaluation among Pauwels’ classification.

	Pauwels I (n = 22)	Pauwels II (n = 167)	Pauwels III (n = 65)
CEA, °	27.9 (6.9)	29.4 (7.7)	30.0 (7.2)
Sharp angle, °	43.0 (5.1)	40.9 (4.6)	40.6 (41)
ARO, °	9.6 (5.4)	9.3 (6.0)	7.9 (5.5)
Acetabular version, °	21.9 (5.9)	20.5 (6.8)*	18.0 (6.2)*
Diameter of acetabulum, mm	51.0 (4.8)	50.3 (3.5)	51.2 (3.5)
Diameter of femoral head, mm	44.0 (4.5)	44.0 (3.5)	44.6 (3.2)

Data are presented as mean (standard deviation). CEA center edge angle, ARO acetabular roof obliquity. *p < 0.05 indicates a statistically significant difference compared to Pauwels I group.

### Univariate and multivariate analyses of Pauwels I and III fractures

Univariate analysis demonstrated that DDH was significantly associated with Pauwels type I fractures. Garden classification showed a strong association with Pauwels classification; however, it was treated as a fracture-related covariate rather than an independent predictor. In contrast, female sex, Garden classification, absence of DDH, and reduced AV were significantly associated with a higher risk of Pauwels type III fractures. Multivariate logistic regression analysis, adjusted for age, sex, BMI, and Garden classification, confirmed that DDH was independently associated with a higher likelihood of Pauwels type I fractures, while the absence of DDH and decreased AV were independently associated with an increased risk of Pauwels type III fractures (Table 4).

**Table 4:** Odds ratios for Pauwels I and III fractures in all patients.

	Univariate analysis			Multivariate analysis *		
	OR	95% CI	P Value	OR	95% CI	P Value
Pauwels I fractures						
Age	0.99	0.95–1.03	0.64			
Sex (female)	1.37	0.50–3.41	0.52			
BMI	1.00	0.89–1.09	0.96			
Garden classification (displaced)	0.21	0.09–0.53	< 0.01			
DDH	3.03	1.22–7.44	0.02	3.76	1.36–10.49	0.01
Acetabular version angle	1.05	0.98–1.13	0.16	1.05	0.98–1.13	0.17
Diameter of acetabulum	1.03	0.91–1.16	0.60	1.13	0.90–1.43	0.29
Diameter of femoral head	0.99	0.87–1.12	0.90	0.84	0.64- 1.10	0.21
Pauwels III fractures						
Age	1.01	0.98–1.03	0.71			
Sex (female)	2.77	1.30–6.64	0.01			
BMI	0.98	0.91–1.05	0.66			
Garden classification (displaced)	23.65	5.01–422.90	< 0.01			
DDH	0.43	0.19–0.91	0.03	0.42	0.17–0.96	< 0.05
Acetabular version angle	0.94	0.90–0.98	< 0.01	0.94	0.90–0.99	0.01
Diameter of acetabulum	1.06	0.98–1.15	0.14	1.10	0.93–1.30	0.25
Diameter of femoral head	1.05	0.97–1.14	0.21	1.04	0.86–1.25	0.70

OR; odds ratio, CI; confidence interval, BMI; body mass index, DDH; developmental dysplasia of the hip. * Adjusted for age, sex, BMI, and Garden classification. Variables were selected based on clinical relevance and minimized through the Akaike Information Criterion (AIC).

## Discussion

The primary objective of this study was not to evaluate treatment strategies or postoperative outcomes, but to clarify the morphological mechanisms underlying fracture configuration in femoral neck fractures. Specifically, we focused on whether pelvic morphology, particularly DDH, is associated with variations in Pauwels classification. To the best of our knowledge, this is the first multicenter study to specifically examine the association between pelvic morphology and Pauwels classification within femoral neck fractures. Previous studies have explored the influence of hip morphology on hip fracture patterns; however, most have focused on distinguishing between femoral neck and trochanteric fractures rather than examining variations within femoral neck fractures themselves. Prior research has largely emphasized femoral morphology, reporting that an increased neck-shaft angle and elongated femoral neck are associated with a higher incidence of femoral neck fractures than trochanteric fractures^10–14^. In patients with a large neck-shaft angle, Rafferty et al. noted that the superior aspect of the femoral neck exhibited reduced cortical thickness, increasing vulnerability to femoral neck fractures^15^.

**Fig. 3:**
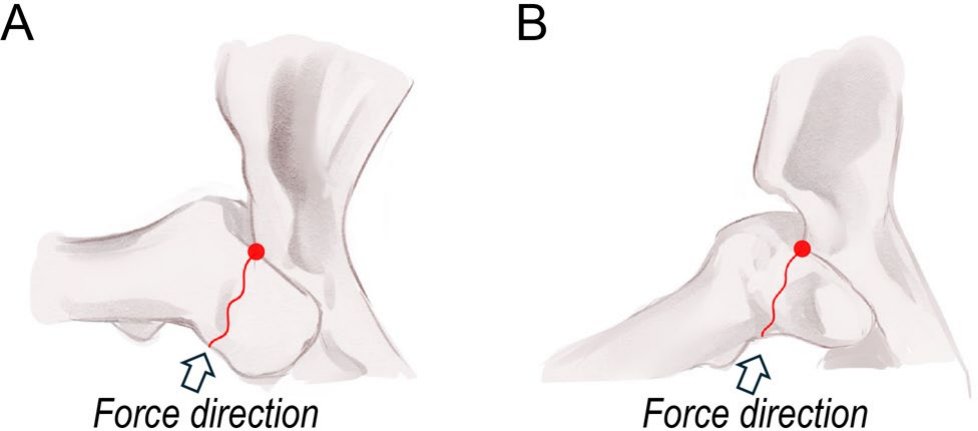
Schematic representation of the impingement mechanism between the femoral neck and acetabular rim during a sideways fall. (**A**) In hips with reduced acetabular coverage (DDH), the effective fulcrum and force transmission across the femoral neck may favor a more horizontal fracture line, consistent with a Pauwels type I pattern. (**B**) In hips without DDH, particularly in those with reduced acetabular version, the force direction across the femoral neck may become steeper, which may contribute to a more vertical fracture line consistent with a Pauwels type III pattern. The red dot indicates a representative contact point and does not necessarily reflect the precise site of fracture initiation. This figure is intended as a conceptual schematic and not as a direct representation of the actual fracture process.

Conversely, several studies have examined the role of acetabular morphology, reporting that patients with femoral trochanteric fractures tend to have a greater CEA than those with femoral neck fractures^10,16^. As the CEA increases, often due to osteophyte formation, the pattern of compressive force transmission across the proximal femur may be altered, which may increase stress on the lateral aspect of the proximal femur during a fall and thereby predispose to trochanteric fractures^10^. A similar mechanism may explain the findings of the present study, which demonstrated a significant association between DDH and Pauwels type I fractures. In hips with reduced acetabular coverage, the effective fulcrum and force transmission across the femoral neck during a sideways fall may favor a more horizontal fracture line, consistent with Pauwels type I. Conversely, in hips without DDH, differences in acetabular coverage and version may alter the effective fulcrum and the direction of force transmission across the femoral neck, which may contribute to a steeper fracture angle and increase the likelihood of Pauwels type III fractures (Fig. 3). Regarding other morphological features, acetabular and femoral head diameters were significantly smaller in the DDH group compared to the non-DDH group. However, these absolute differences were modest (approximately 1 mm) and were not identified as independent predictors of Pauwels classification in the multivariate analysis. Furthermore, considering that the DDH group contained a significantly higher proportion of female patients, these diameter differences likely reflect variations in sex and body size rather than being primary morphological factors determining fracture patterns. Therefore, while smaller joint diameters are a recognized feature of the DDH phenotype, they should be interpreted as associated characteristics rather than key drivers of fracture configuration.

One of the important findings of this study is the significant reduction in AV observed in Pauwels type III fractures. While previous studies have linked acetabular retroversion to femoral neck stress injuries under repetitive loading, our data suggest that such morphological variations may also influence the configuration of acute fractures. However, this connection is indirect, as acute low-energy trauma involves a single impact mechanism rather than chronic impingement. Therefore, we place greater emphasis on our biomechanical hypothesis based on the shift of the contact point. Specifically, the shift in the contact point between the femoral neck and the acetabular rim may occur in three dimensions rather than along a single anatomical axis. In our cohort, the Pauwels type III group demonstrated not only decreased AV but also a significantly smaller CEA. These findings indicate a combined reduction in both anterior (associated with decreased AV) and lateral (associated with smaller CEA) acetabular coverage, rather than a proximal migration of the femoral head. This combined reduction in acetabular coverage may shift the contact point between the femoral neck and the acetabular rim distally and laterally during a fall. As a result, the direction and magnitude of shear forces acting across the femoral neck may change, potentially promoting a more vertically oriented fracture line consistent with a Pauwels type III configuration.

Postoperative outcomes were not evaluated in the present study; however, our findings provide a potential morphological explanation for variations in fracture configuration. Accordingly, these results should be regarded as hypothesis-generating rather than directly practice-changing. For example, in DDH patients presenting with Pauwels type I fractures, acetabular morphology may warrant consideration of valgus osteotomy or arthroplasty, particularly when femoroacetabular impingement is suspected. Thus, pelvic morphology assessment may serve as a radiological adjunct in choosing surgical strategies, which warrants further investigation. The results of this study have important clinical implications. Specifically, clinicians should consider the possibility of underlying acetabular dysplasia in patients presenting with Pauwels type I femoral neck fractures. Previous studies have identified reduced CEA as a risk factor for dislocation following bipolar hemiarthroplasty^17–19^. Therefore, careful assessment of acetabular morphology remains warranted when planning surgical management for Pauwels type I fractures, and total hip arthroplasty should be considered in cases of significant acetabular dysplasia.

This study has some limitations. First, Pauwels’ classification was determined based solely on anteroposterior radiographs according to the original method, which may not fully capture the three-dimensional orientation of the fracture line. Second, our analysis focused exclusively on acetabular morphology without accounting for femoral morphological variations. Third, we did not assess bone mineral density, which may influence fracture patterns. Concerning the second and third limitations, it is technically difficult to evaluate the fracture side, and there is no guarantee that the healthy side has the same shape or density; therefore, it is not appropriate to evaluate as a substitute. Fourth, acetabular version was measured on supine CT images, which may not fully represent functional acetabular orientation in the standing position. However, supine CT measurements remain widely used for assessing acetabular morphology and are considered to reasonably reflect underlying acetabular structure despite postural variation. Fifth, the number of Pauwels type I fractures is relatively small. Because the number of outcome events was limited, the multivariable model may have been susceptible to overfitting. Although the analysis was performed to explore potential associations between acetabular morphology and fracture configuration, these findings should be interpreted cautiously and validated in future studies with larger sample sizes. Despite these limitations, this multicenter prospective cohort study benefits from a relatively large sample size, enhancing the robustness of our findings.

## Conclusion

This study identified a significant association between DDH and Pauwels’ classification in patients with femoral neck fractures. DDH was independently associated with an increased incidence of Pauwels type I fractures and a decreased incidence of Pauwels type III fractures. Furthermore, the acetabular version was significantly reduced in Pauwels type III fractures compared to types I and II, suggesting a potential role of acetabular retroversion in developing more vertically oriented fracture patterns.

## Data Availability

The datasets generated during and/or analysed during the current study are available from the corresponding author on reasonable request.
